# Benefits of Exercise on Influenza or Pneumonia in Older Adults: A Systematic Review

**DOI:** 10.3390/ijerph17082655

**Published:** 2020-04-13

**Authors:** Yang Song, Feng Ren, Dong Sun, Meizi Wang, Julien S. Baker, Bíró István, Yaodong Gu

**Affiliations:** 1 Faculty of Sports Science, Ningbo University, Ningbo 315211, China; NBUsongyang@hotmail.com; 2 Faculty of Engineering, University of Pannonia Veszeprem, 8200 Veszprém, Hungary; nbsundong@gmail.com (D.S.); nbuwangmeizi@aliyun.com (M.W.); 3 Department of Sport and Physical Education, Hong Kong Baptist University, Hong Kong 999077, China; jsbaker@hkbu.edu.hk; 4 Faculty of Engineering, University of Szeged, 6724 Szeged, Hungary; biro-i@mk.u-szeged.hu

**Keywords:** exercise, influenza, pneumonia, older adults

## Abstract

A coronavirus pandemic has recently become one of the greatest threats the world is facing. Older adults are under a high risk of infection because of weaker immune systems. Therefore, the purpose of this review is to summarize the recent scientific evidence that outlines the effects of exercise on influenza or pneumonia in older adults. An electronic literature search was conducted using the WEB OF SCIENCE, SCIENCEDIRECT and GOOGLE SCHOLAR databases using the following keywords, “Exercise,” “Older adult,” “Influenza,” and “Pneumonia.” Any randomized control trials, cross-sectional and observational studies that related to this topic were all included. Twenty studies met the eligibility criteria for this review. Thirteen randomized control trials investigated the effects of exercise on the immune responses to influenza or pneumonia vaccination: seven trials employed moderate aerobic exercise, three employed resistance exercise, and the remaining three used Asian martial arts or special home-based exercises. Five cross-sectional and two observational studies examined the associations between exercise/physical condition and influenza/pneumonia. Most of the current studies suggested that prolonged moderate aerobic exercise may help to reduce the risk of influenza-related infection and improve the immune responses to influenza or pneumonia vaccination in older adults. In addition, training in traditional Asian martial arts was also found to be beneficial. Future research should focus on the different effects of moderate and vigorous exercise on influenza-related diseases.

## 1. Introduction

Virus infection has had a profound effect on human history and mortality. One of the deadliest viruses documented in human history is pandemic influenza virus [1]. Although influenza is a common respiratory disease, several serious complications, such as pneumonia, could develop if the disease is not controlled rapidly and effectively [2]. It has been estimated that seasonal influenza may cause 3–5 million cases of severe illness, and lead to almost 500,000 deaths annually because of the increasing susceptibility in populations [3]. In fact, the four main influenza pandemics over the last hundred years, which occurred in 1918, 1957, 1968, and 2009 respectively, caused millions of deaths across the world [4,5,6].

Recently, a novel coronavirus disease, officially known as corona virus disease 2019 (COVID-19), is posing a great threat to human life and property [7]. Initial outbreaks were first recorded in Wuhan, a major city in mainland China, in December 2019, and now the disease has spread rapidly all over the world. According to the official website of National Health Commission of the People’s Republic of China, the coronavirus pneumonia had killed 3318 and infected 81,589 people in China by 24:00, 1 April 2020 (Local time). In addition, there were 20,072 people under risk of infection because of close contact with infected individuals [8]. At the same time, 823,626 cases of severe acute respiratory syndrome coronavirus 2 (SARS-CoV-2) infection had been detected and confirmed in more than 100 countries based on the latest World Health Organization (WHO) report (10:00 CET 1 April 2020) [9]. Concerns about COVID-19 have not reduced, and the United States is fighting with a recent wave of winter influenza. According to the latest data from the United States Center for Disease Control and Prevention (CDC), at least 32 million people in the United States have been infected with the flu virus since the 2019–2020 winter flu season, and influenza-related deaths could reach to 18,000, and is arguably one of the deadliest flu outbreaks in the past 40 years [10]. Therefore, increasing vaccine research and exploring other methodologies that may provide beneficial effects to improve protection from the disease is of great clinical importance globally.

Previous studies have indicated that moderate exercise is associated with several anti-influenza benefits, including the reduction of influenza risk, and increased rates of vaccine efficacy [2,7,11,12,13]. For example, Kohut et al. [2] evaluated the effects of moderate exercise on antibody responses to influenza vaccination in older adults, and they found that an exercise group presented a significantly greater increase in antibody titer. Moreover, an animal study conducted by Lowder et al. [11] investigated the effects of different doses of exercise on immune responses, morbidity, and mortality after mice has been infected by influenza virus. The results showed that moderate intensity exercise can significantly reduce the mortality of infected mice. However, contrary findings to this also exist. Ranadive et al. [14] studied the effects of acute moderate exercise on influenza vaccine effectiveness, suggesting that acute moderate exercise does not enhance the vaccine efficacy in older men. Similarly, Long et al. [15] also reported that moderate intensity brisk walking has no effect on antibody responses to both influenza and pneumonia immunization. The literature suggests that physical exercise may exert some benefits on the prevention and treatment of influenza-related diseases, an extensive review, however, is required for further verification.

Virtually every country in the world is experiencing growth in the number and proportion of older persons in their population [3]. By 2050, 21.1% of the world population will be 60 years or older, which is unprecedented in human history [16,17]. Meanwhile, the rapid increase in aged populations poses a great threat to the healthcare system [3]. Aging is inevitably associated with declining organic and physiological function, such as immunosenescence, which refers to the age-related decline in immune capacity [18,19]. There is sufficient clinical evidence to demonstrate that older adults are more vulnerable to infectious diseases (especially influenza, pneumonia) and also have lower immune responses to vaccines in relation to younger or middle-aged counterparts [15,20,21,22,23]. For example, Long et al. [15] reported that older individuals had greater lower antibody responses to some of the influenza and pneumonia vaccination strains when compared to younger individuals. Hence, considering the potential benefits of exercise, there is an urgent need to explore effective exercise strategies to facilitate healthy aging.

In this review, we aim to summarize the current literature regarding the effects of exercise on influenza or pneumonia in older adults, and determine the appropriate exercise form that contributes to beneficial clinical outcomes in old populations.

## 2. Methods

### 2.1. Eligibility Criteria

The inclusion criteria for screening articles included the choice of subjects, experimental design, and outcome analysis. (1) Subjects: only studies related to older adults were included in this review, it was also acceptable if the subjects in the studies were of poor physical condition; (2) experimental design: any clinical comparative research or correlational studies regarding exercise, influenza, and/or pneumonia in older adults were all included in the study; (3) outcome analysis: the results analysis was aimed at investigating the effects of exercise on influenza or pneumonia, or the association between exercise/physical condition and influenza or pneumonia in older adults. In addition, the exclusion criteria were that, studies met the inclusion criteria but were then excluded because of duplicates between databases or relative conditions.

### 2.2. Search Strategy

To ensure a non-biased and complete review, a thorough literature search was conducted using the following three large electronic databases: WEB OF SCIENCE (1960–present), SCIENCEDIRECT (all years), and GOOGLE SCHOLAR (all years). The final literature search was performed on 13 February 2020. Four keywords were employed for the literature search in each database, “exercise,” “older adult,” “influenza,” and “pneumonia.” In the WEB OF SCINECE database, the basic search in all databases was chosen and keywords were entered in order: “exercise” AND “older adult” AND “influenza” OR “pneumonia.” In the SCIENCEDIRECT database, we chose a keyword search with keywords: “exercise” AND “older adult” AND “influenza” OR “pneumonia.” In GOOGLE SCHOLAR, an “advanced search” was used, “exercise” and “older adult” were put into “with all of the words” option while “influenza” and “pneumonia” in “with at least one of the words” option, and “anywhere in the article” were chosen in the option of “where my words occur.”

To ensure the final number of studies included in this review, all the retrieved articles were independently screened and assessed by two authors and if there were any differences regarding inclusion, a third author was invited to join the discussion and make a decision. In addition, reference lists of retrieved review articles and the eligible studies were further checked using the snowballing method to ensure that no paper has been potentially overlooked [24]. These studies were subsequently searched to obtain full texts based on titles and authors.

### 2.3. Data Extraction

**Figure 1:**
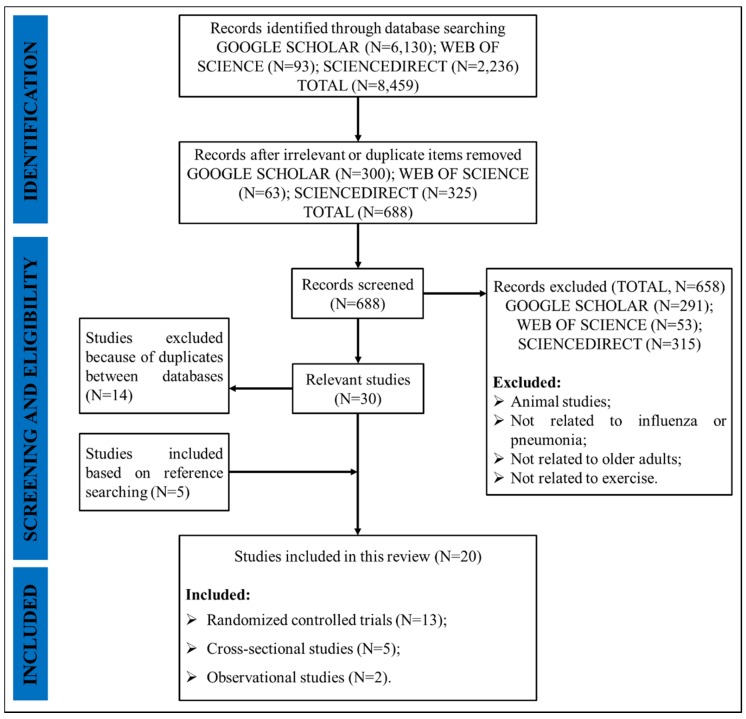
The search flowchart.

The search procedure is presented in Figure 1. A total of 8459 articles were searched from the three databases and articles were reduced to 688 after all the duplicate and irrelevant records were excluded via checking the title and/or abstract. Based on the inclusion and exclusion criteria, 53 papers from WEB OF SCIENCE, 315 papers from SCIENCEDIRECT, and 291 papers from GOOGLE SCHOLAR were further excluded. Five additional studies were found after checking the reference lists. However, 14 articles were duplicates between databases. Thus, 20 studies met the inclusion criteria for this review. The following data were extracted and summarized from the final included studies: authors, nationality of the first author, published year, published journal, subjects’ number and basic information, objectives, study design, and main findings. Mendeley Reference Management Software (Elsevier Ltd., Amsterdam, The Netherlands) was used for organizing the papers and generating citations.

## 3. Results

Twenty trials met the eligibility criteria and were included in this review. The study characteristics were summarized and presented in two parts: (1) Randomized controlled trials (RCT) regarding the effects of exercise on influenza or pneumonia (13 studies, Table 1); (2) cross-sectional (X-Sec) or observational (Obs) studies regarding the association between exercise/physical condition and influenza or pneumonia (7 studies, Table 2). In addition, the main findings of these papers are summarized in Table 3.

### 3.1. Randomized Controlled Trials

#### 3.1.1. Moderate Aerobic Exercise

The majority of RCT (seven of thirteen trials) investigated the effects of moderate aerobic exercise (brisk walking, treadmill running, and stair climbing etc.,) on the immune responses to influenza or pneumonia vaccination. Kohut and colleagues first started RCT on older adults [2,12], they employed the same moderate aerobic exercise task in two papers. Subjects were asked to take part in an aerobic exercise training regime three times per week for 10 months in total. They used treadmill, cycle ergometers, and other equipment for exercise modalities at an intensity of 65%–75% heart rate reserve for 25–30 min per exercise session. The research mainly evaluated the effects of chronic moderate exercise on the antibody responses to influenza vaccination while the second study further investigated the role of psychosocial factors in the process. Both studies found that long duration moderate aerobic exercise training significantly improved the immunocompetence of older individuals, and they speculated that the endogenous opioids may possibly help to mediate the exercise-induced alterations. Moreover, the second study further inferred that psychological factors could be another possible mechanism.

Similarly, two subsequent studies based on chronic moderate aerobic exercise also reported positive effects. In order to eliminate the impact of prior vaccine experience on immune responses, Vieira et al. [31] further compared the effects of a 10-month cardiovascular exercise on antibody responses in older adults with and without vaccine history respectively. They found that subjects with previous vaccine experience exhibited higher hemagglutination inhibition (antibody titer) 24 weeks after exercise. Wood et al. [13] reported that there were significant improvements in influenza seroprotection, overall illness severity and sleep disturbance in older adults who had completed a 10-month brisk walking exercise program when compared with those involved in balance and flexibility training. They demonstrated that the reduced inflammatory biomarkers or memory to naïve T lymphocyte ratio may contribute to the benefit of chronic moderate aerobic exercise on immune responses, although it has not been tested in their study.

However, more recently, three studies have proposed some conflicting and equivocal results. The first of three studies aimed to investigate the different effects of meditation and exercise on influenza vaccine protection. Subjects were randomly assigned to moderate sustained exercise or meditation training for 8 weeks. The results showed no differences in antibody responses were found either after meditation or exercise intervention [28]. Therefore, they hypothesized that the exercise-induced benefits may be associated with innate immune processes, or have not been detected because of limited parameters being assessed. Two additional studies used a similar single bout of moderate aerobic exercise intervention. Long et al. [15] examined whether acute moderate exercise can improve immune responses to pneumonia and half dose influenza immunization and found no enhanced responses to either of the vaccines. Ranadive et al. [14] also reported no difference in antibody responses in older men. However, results were not uniform in men and women. They found that acute moderate aerobic exercise can significantly enhance the immune responses to full dose influenza vaccination in older women. Although both studies reported no improvements after exercise (except women in Ranadive et al. study [14]), they all suggested that interleukin-6 could be the possible biomarker which may help the antibody response.

#### 3.1.2. Resistance Exercise

Three studies investigating the effects of prolonged or acute resistance exercise on influenza or pneumonia were included in this review. Dangour et al. [26] reported that either a nutritional program or resistance exercise, and even nutritional program with resistance exercise was not effective in reducing the risk of pneumonia infection. Also, it is interesting to note that, no significant effect was found even after including aerobic exercises (e.g., dancing, ball games) into the exercise program under participants’ requests. However, they stated that the insufficient statistical power may partly result in the non-significant results. The other two studies employed a similar exercise task, and both demonstrated that acute resistance exercise did not enhance the immune responses to influenza vaccination [25,27]. Bohn-Goldbaum et al. [25] further discussed the internal mechanism, and they speculated that the relatively longer duration of resistance exercise used in their study may decrease the lymph flow rate, which then weakens the adjuvant effect.

#### 3.1.3. Other Exercises

There are also three studies that investigated the effects of traditional Asian martial arts (e.g., Taiji, Qigong) or special home-based exercise on influenza or pneumonia in older adults. The first of three studies investigated whether 5-month Taiji and Qigong practice can help to enhance the antibody responses to influenza vaccination [32]. The results showed that long-term moderate Taiji and Qigong practice exhibited higher hemagglutination inhibition titers throughout the whole period, suggesting that the prolonged practice of Taiji and Qigong does enhance the immune response. In addition, two recent studies developed a similar home-based exercise for aspiration pneumonia prevention and falls [29,30]. Although one study did not specify the duration of the intervention, both reported that such exercise did help prevent aspiration pneumonia and falls. In summary, these exercise forms are novel and effective, and the effectiveness and potential mechanisms should be further investigated.

### 3.2. Observational or Cross-Sectional Trials

Of the remaining seven studies concentrating on the association between exercise/physical condition and influenza or pneumonia, five were X-sec studies, assessing the fitness status of participants by physical activity questionnaire and/or maximal oxygen uptake test. All the studies reported significantly positive associations between exercise/physical condition and influenza-related mortality or immunocompetence. However, the recommended exercise intensity may vary between studies. After influenza vaccination, both Schuler et al. [37] and Keylock et al. [34] found positive results in physically fit older adults, suggesting that regular exercise (intensity not specified) can significantly enhance the antibody responses to influenza vaccination in older individuals. Similarly, Wong et al. [38] investigated the association between exercise and influenza-induced morality in 24,656 adults who died in 1998 in Hong Kong and reported that exercising once per month to three time per week (intensity not specified) contributed to lowest morality. In 2002, a study by Kohut et al. [35] demonstrated that only regular, vigorous aerobic exercise contributed to greater immune responses to influenza vaccination when compared with moderate exercise and sedentary behavior. However, a recent study by de Araújo et al. [33] reported that both moderate and vigorous exercise exhibited higher antibody responses.

In addition, there are two observational studies that examined the effects of physical condition (frail and non-frail). Subjects were classified as physically frail or non-frail by the standard frailty criteria, including endurance, strength, physical activity etc., [40]. Yao et al. [39] reported that frailty can significantly impair the immune responses and increase the rate of influenza-like illness. Moehling et al. [36] compared the effects of frailty and non-frailty on immunocompetence in subjects aged 50–65 respectively. Interestingly, the results showed that frail individuals aged 50–65 had higher immune responses to influenza vaccination while there was no difference in the non-frail group.

## 4. Discussion

This literature review summarized studies examining the effects of exercise on influenza or pneumonia, the association between exercise/physical condition and influenza or pneumonia, with the aim to determine the appropriate exercise duration and type that can contribute to beneficial clinical outcomes in old populations.

The findings from RCT suggest that prolonged moderate aerobic exercise, rather than single-dose exercise, enhances the immune responses to influenza and pneumonia vaccination. Two studies in this review employed a single bout of moderate aerobic exercise and found no significant differences in immunocompetence when compared to a control group [14,15]. However, a female population study by Ranadive et al. [14] recorded an enhanced response after exercise. Further studies should consider the potential effects of sex differences. However, a review by Psdcoe et al. [41] aimed to examine the effects of both acute and chronic exercise on the immune responses to vaccination and demonstrated that acute moderate-intensity exercise (one bout employed at the time of vaccination) may also contribute positively to the antibody responses. Explanation for the lack of consistency for this study could be the age difference. Most studies that reported similar findings focused on the effects of exercise on immunocompetence in young subjects [42,43,44,45]. However, although all the X-sec studies demonstrated positive associations between exercise and influenza-related mortality or immunocompetence, there seems to be no consistent intensity of exercise reported as both moderate and vigorous exercise may boost the antibody responses [33,35]. More research comparing the effects of moderate and intensive exercise needs completion for further verification.

Taiji practice, a traditional Asian martial art, combines Daoist philosophy, Qigong (meditation) and Chinese medicine, and focuses on the balance between strength and dynamic stability [32]. It is worth noting that one study in this review found that Taiji practice can enhance the immune responses to influenza immunization [32]. More studies are needed to further ensure its effectiveness and potential mechanisms. The special home-based exercise program also exhibited preventive effects to aspiration pneumonia [29,30]. However, this kind of exercise form may relate to disease types and thus a lack of generalization is probable. Physical frailty, a concept that can be applied to evaluate physical fitness, is marked by diminished endurance, strength, and decreased physical function [36,46]. Two studies that examined the effects of physical frailty on immunocompetence were included in this review as this may also reflect the role of exercise (physical condition) [36,39]. However, the results appeared to be conflicting. One study by Yao et al. [39] reported that physical frailty may impair the immune responses and increase the rate of influenza-like illness while Moehling et al. [36] found that subjects aged 50–65 with physical frailty exhibited significantly greater immune responses, which is completely opposite to the results of all the other observational and X-sec studies. However, the underlying cause was not further investigated in their studies.

The possible underlying mechanisms behind the exercise-induced beneficial effects on influenza (or pneumonia) vaccination (or prevention) have been widely speculated previously, but it becomes restricted after these included studies being divided into several groups because of different exercise interventions. Prolonged moderate aerobic exercise has been previously proven and further confirmed in this review that it contributes some positive benefits on influenza (or pneumonia) immunization. The role of endogenous opioids, inflammatory biomarkers, or memory to naive T lymphocyte ratio have received some support, suggesting they may be associated with the subsequent exercise-induced benefits. Interestingly, Kohut et al. [12] demonstrated that the mechanisms of exercise-induced enhancement in immune responses may also involve psychological factors (e.g., depression, stress). Indeed, there are some studies that have concentrated on the effects of psychology on immunocompetence and an additional review could help to elucidate these outcomes.

Although two studies that examined the effect of acute aerobic exercise found no significant improvement, they inferred that more vigorous exercise may augment the antibody response by elevating the interleukin-6 circulating level, which has been noted as the potential mechanism for acute aerobic exercise-induced improvement. A review conducted by Psdcoe et al. [41] also supported the above hypothesis. In addition, the mechanisms by which exercise improves immune response may vary with exercise forms. Bohn-Goldbaum et al. [25] demonstrated that the lymph flow rate, rather than interleukin-6 circulating levels, could be one of the biomarkers for the enhancement stimulated by acute resistance exercises. Yang et al. [32] inferred that the changes of autonomic balance induced by Taiji and Qigong practice may be the potential mechanism for the enhanced responses although it has not been tested. In summary, while the internal mechanisms remain inconclusive, it is well-established that there are several links between neuroendocrine factors and exercise that could impact positively on the cells of the immune system [47,48,49,50,51,52,53]. More physiological studies are much warranted to further reveal the associated mechanistic biochemistry.

## 5. Conclusions

In conclusion, this literature review confirms that long-duration moderate aerobic exercise may help reduce the risk of influenza-related infection, improve the immune responses to influenza and pneumonia vaccination in older adults. In addition, traditional Asian martial arts may also contribute some related benefits. However, because of the lack of studies, some areas need further consideration, including the different effects of moderate and intensive exercise on influenza or pneumonia.

## Figures and Tables

**Table 1:** The study characteristics of randomized controlled trials.

Author and Year	Country	Disease	Sample Size (N)	Age (year) and Gender	Exercise
Bohn-Goldaum et al. (2019) [25]	Australia	Influenza	N = 46 EG = 23 CG = 23	Age: EG = 74.4 ± 6.5; CG = 72.3 ± 6.7 Gender: EG, M = 11, F = 12; CG, M = 11, F = 12	EG: Resistance exercise CG: Rest
Dangour et al. (2011) [26]	Britain	Pneumonia	N = 1500 EG = 480 NS + EG = 516 CG = 504	Age: EG = 66.1 ± 0.9; NS + EG = 66.2 ± 1.0; CG = 66.1 ± 1.0 Gender: EG, M = 141, F = 339; NS + EG, M = 163, F = 353; CG, M = 186, F = 318	EG: Resistance exercise + recreational activities NS + EG: Resistance exercise + recreational activities + nutritional supplement CG: Rest
Edwards et al. (2015) [27]	Australia	Influenza	N = 46	Age: 73 ± 7 Gender: M = 23, F = 23	EG: Resistance exercise CG: Rest
Hayney et al. (2014) [28]	USA	Influenza	N = 98 EG = 47 CG = 51	Age: EG = 59.0 ± 6.6; CG = 58.8 ± 6.8 Gender: EG, M = 8, F = 39; CG, M = 10, F = 41	EG: Sustained exercise CG: Rest
Kohut et al. (2004) [2]	USA	Influenza	N = 27 EG = 14 CG = 13	Age: EG = 73.07 ± 5.6; CG = 70.25 ± 5.6 Gender: N/A	EG: Aerobic exercise CG: Low intensity exercise or rest
Kohut et al. (2005) [12]	USA	Influenza	N = 27 EG = 14 CG = 13	Age: EG = 70.25 ± 5.57; CG = 73.07 ± 5.59 Gender: EG, M = 7, F = 7; CG, M = 6, F = 7	EG: Aerobic exercise CG: Walking or rest
Long et al. (2012) [15]	Britain	Influenza + Pneumonia	N = 62 EG = 31 CG = 31	Age: EG = 57.94 ± 4.40; CG = 58.55 ± 4.38 Gender: N/A	EG: Aerobic exercise CG: Rest
Matsumoto et al. (2015) [29]	Japan	Pneumonia	N = 208 EG = 137 CG = 71	Age: EG = 74.5 ± 5.6; CG = 74.9 ± 6.6 Gender: EG, M = 35, F = 102; CG, M = 13, F = 58	EG: Home-based exercise with expiratory muscle training CG: Rest
Ranadive et al. (2014) [14]	USA	Influenza	N = 55 EG = 28 CG = 27	Age: EG = 66 ± 0.93; CG = 67 ± 0.77 Gender: N/A	EG: Aerobic exercise CG: Rest
Takatori et al. (2016) [30]	Japan	Pneumonia	N = 266 EG = 148 CG = 118	Age: EG = 74.6 ± 5.1; CG = 75.9 ± 6.0 Gender: F = 266	EG: Specifically home-based exercise programme CG: General stretching exercise
Vieira et al. (2008) [31]	USA	Influenza	N = 145 EG = 75 CG = 70	Age: 60–83 Gender: N/A	EG: Cardiovascular exercise CG: Flexibility or balance exercise
Woods et al. (2009) [13]	USA	Influenza	N = 144 EG = 74 CG = 70	Age: EG = 69.6 ± 4.9; CG = 70.1 ± 5.7 Gender: EG, M = 27, F = 47; CG, M = 27, F = 43	EG: Cardiovascular exercise CG: Stretching and balance exercise
Yang et al. (2007) [32]	USA	Influenza	N = 50 EG = 27 CG = 23	Age: EG = 79.5 ± 1.9; CG = 74.1 ± 2.0 Gender: EG, M = 6, F = 21; CG, M = 7, F = 16	EG: Qigong and Taiji form practice CG: Rest

Note: exercise group, EG; control group, CG; nutritional supplement, NS; male, M; female, F.

**Table 2:** The study characteristics of cross-sectional and observational studies.

Author and Year	Country	Disease	Sample Size (N)	Age (year) and Gender
de Araújo et al. (2015) [33]	Brazil	Influenza	N = 61 Intense exercise = 22 Moderate exercise = 23 Never exercise = 16	Age: Intense exercise = 74.8 ± 1.5; Moderate exercise = 70.4 ± 0.7; Never exercise = 72.9 ± 1.5 Gender: M = 61
Keylock et al. (2007) [34]	Korea	Influenza	N = 26 Physically active, high-fit = 13 Sedentary, low-fit = 13	Age: Physically active, high-fit = 64.8 ± 1.2; Sedentary, low-fit = 67.9 ± 1.2 Gender: M = 13, F = 13
Kohut et al. (2002) [35]	USA	Influenza	N = 56 Active = 16 Moderately active = 25 Sedentary = 15	Age: Active = 71.9 ± 5.2; Moderately active = 70.7 ± 6.3; Sedentary = 71.5 ± 7.1 Gender: Active, M = 7, F = 9; Moderately active, M = 8, F = 17; Sedentary, M = 6, F = 9
Moehling et al. (2017) [36]	USA	Influenza	N = 114 Group1: Non-frail = 37, Frail = 29 Group2: Non-frail = 22, Frail = 18	Age: Group1: Non-frail = 56.1–61.0, Frail = 54.4–61.8; Group2: Non-frail = 66.6–73.6, Frail = 68.1–74.0 Gender: Group1: Non-frail, M = 9, F = 28; Frail, M = 6, F = 23; Group2: Non-frail, M = 5, F = 17; Frail, M = 6, F = 12
Schuler et al. (2003) [37]	USA	Influenza	N = 30	Age: 81 ± 5; Gender: M = 10; F = 20
Wong et al. (2008) [38]	Hong Kong, China	Influenza	N = 24,656 Frequent exercise = 4787 Low/moderate exercise = 2852 Never exercise = 16,414	Age: 30–64 (21%), 65-(79%) Gender: N/A
Yao et al. (2011) [39]	USA	Influenza	N = 71 Non-frail = 22 Pre-frail = 32; Frail = 17	Age: Non-frail = 82.0 ± 5.4; Pre-frail = 85.4 ± 4.1; Frail = 86.0 ± 3.1 Gender: Non-frail, M = 4; F = 18; Pre-frail, M = 10; F = 22; Frail, M = 2; F = 15

**Table 3:** The main findings of studies.

Author & Year	Study Design and Intervention	Primary Results
**Clinical Comparative Studies**		
Bohn-Goldaum et al. (2019) [25]	RCT EG: Resistance exercise: 5 separate resistance exercise, 45 min (8 repetition, 60% of 1RM, 2 min recovery for each exercise) CG: Rest, 45 min	(1) No significant differences between groups over 6 months for antibody response to influenza immunization.
Dangour et al. (2011) [26]	RCT EG: Resistance exercise: chair stands, modified squats etc., 24 months (1 h × 2 times/week) NS+EG: Nutritional supplement + Resistance exercise, 24 months CG: Rest, 24 months	(1) No significant differences between groups for the incidence rate of pneumonia at 24-month time point.
Edwards et al. (2015) [27]	RCT EG: Resistance exercise: upper and lower body muscle groups (60% of 1 RM, 1 time) CG: Rest	(1) No significant differences between groups at baseline, 1 or 6-month time points for antibody response to influenza immunization.
Hayney et al. (2014) [28]	RCT EG: Group exercise + home exercise, 8 weeks (2.5 h/week + 45 min/day) CG: Rest, 8 months	(1) No significant differences between groups over 8 months to influenza immunization. (2) Psychological states may correlate with antibody responses.
Kohut et al. (2004) [2]	RCT EG: Aerobic exercise: treadmills, stair-steppers etc., 10 months (65%–75%HHR, 25–30 min × 3 times/week) CG: Low intensity exercise or rest, 10 months	(1) EG have significantly greater antibody response to influenza immunization.
Kohut et al. (2005) [12]	RCT EG: Aerobic exercise: treadmills, cycle ergometer etc., 10 months (65%–75% HR, 25–30 min × 3 times/week) CG: Low intensity exercise: walking or rest, 10 months	(1) EG have significantly greater antibody response to influenza immunization. (2) Psychological states also involved in improving immune-competence.
Long et al. (2012) [15]	RCT EG: Aerobic exercise: brisk walk, 45 min (At or above 55% HR) CG: Rest, 45 min	(1) No significant differences between groups for antibody response to influenza and pneumonia immunization at four-week time point.
Matsumoto et al. (2015) [29]	RCT EG: Home-based exercise guidance: stretching, muscle training etc. CG: Rest	(1) EG have significantly greater effects for prevention of aspiration pneumonia.
Ranadive et al. (2014) [14]	RCT EG: Aerobic exercise, 40 min (55%–65% HR) CG: Rest, 40 min	(1) No significant differences between groups for antibody response to influenza immunization, except women in the EG.
Takatori et al. (2016) [30]	RCT EG: Home-based exercise programme: stretching, muscle strengthening etc., 6 months (5 min × 3 times/week) CG: General stretching exercises, 6 months	(1) EG have significantly greater effects for prevention of aspiration pneumonia.
Vieira et al. (2008) [31]	RCT EG: Cardiovascular exercise, 10 months CG: Flexibility/balance exercise, 10 months	(1) EG with pre-vaccination experiences have significantly greater antibody response to influenza immunization at 24-week time point.
Woods et al. (2009) [13]	RCT EG: Cardiovascular exercise: brisk walking at least 2 times/week, 10 months (60%–70% HR, 45–60 min × 3 times/week) CG: Flexibility and balance exercise, 10 months (75 min × 2 times/week)	(1) EG have significantly greater seroprotection rate to influenza immunization at 24-week time point.
Yang et al. (2007) [32]	RCT EG: Qigong and Taiji form practice, 20 weeks (1 h × 3 times/week) CG: Routine activities, 20 weeks	(1) EG have significantly greater antibody response to influenza immunization at 3, 6, and 20-week time points.
**Correlational Studies**		
de Araújo et al. (2015) [33]	X-Sec International physical activity questionnaire and VO_2_max treadmill consumption test	(1) Both moderate and intense exercise lifestyle contribute to greater antibody response to influenza immunization.
Keylock et al. (2007) [34]	X-Sec VO_2_max treadmill consumption test	(1) High-fit elderly have significantly greater antibody response to influenza immunization.
Kohut et al. (2002) [35]	X-Sec Phone interviews assessing the level of physical activity	(1) Regular, vigorous aerobic exercise is associated with greater antibody response to influenza immunization.
Moehling et al. (2017) [36]	Obs 4-item summed frailty score (weakness, self-reported exhaustion, walking time and physical activity)	(1) Antibody responses to influenza vaccine is greater in non-frail persons > 65, while an opposite results occurred in persons between 50–65 years old.
Schuler et al. (2003) [37]	X-Sec Physical activity scale for the elderly	(1) Positive correlation between physical activity and antibody response to influenza immunization.
Wong et al. (2008) [38]	X-Sec Physical activity questionnaire (ten years before deaths)	(1) Low to moderate exercise (1 time/month to 3 times/week) contributes to lowest influenza-associated mortality.
Yao et al. (2011) [39]	Obs Validated set of frailty criteria	(1) Non-frail older adults have greater antibody response to influenza vaccine and lower rates of influenza infection.

Note: Randomized Controlled Trial, RCT; Cross-sectional, X-Sec; Observational, Obs; Heart Rate Reserve, HHR; Heart Rate, HR; Exercise Group, EG; Control Group, CG; Nutritional Supplement, NS.

## References

[1] Kain T., Fowler R. (2019). Preparing intensive care for the next pandemic influenza. Crit. Care.

[2] Kohut M.L., Arntson B.A., Lee W., Rozeboom K., Yoon K., Cunnick J.E., McElhaney J. (2004). Moderate exercise improves antibody response to influenza immunization in older adults. Vaccine.

[3] Cao W., Kim J.H., Chirkova T., Reber A.J., Biber R., Shay D.K., Sambhara S. (2011). Improving immunogenicity and effectiveness of influenza vaccine in older adults. Expert Rev. Vaccines.

[4] Johnson N.P.A.S., Mueller J. (2002). Updating the accounts: Global mortality of the 1918–1920 ‘Spanish’ influenza pandemic. Bull. Hist. Med..

[5] Belshe R.B. (2005). The origins of pandemic influenza—Lessons from the 1918 virus. N. Engl. J. Med..

[6] Guan Y., Vijaykrishna D., Bahl J., Zhu H., Wang J., Smith G.J.D. (2010). The emergence of pandemic influenza viruses. Protein Cell.

[7] Zhu W. (2020). Should, and how can, exercise be done during a coronavirus outbreak? An interview with Dr. Jeffrey A. Woods. J. Sport Health Sci..

[8] National Health Commission of the People’s Republic of China Apr 1: Daily Briefing on Novel Coronavirus Cases in China. http://en.nhc.gov.cn/2020-04/02/c_78679.htm.

[9] World Health Organization Coronavirus Disease 2019 (COVID-19) Situation Report-72. https://www.who.int/docs/default-source/coronaviruse/situation-reports/20200401-sitrep-72-covid-19.pdf?sfvrsn=3dd8971b_2.

[10] Centers for Disease Control and Prevention Weekly U.S. Influenza Surveillance Report. https://www.cdc.gov/flu/weekly/index.htm.

[11] Lowder T., Padgett D.A., Woods J.A. (2005). Moderate exercise protects mice from death due to influenza virus. Brain Behav. Immun..

[12] Kohut M.L., Lee W., Martin A., Arnston B., Russell D.W., Ekkekakis P., Yoon K.J., Bishop A., Cunnick J.E. (2005). The exercise-induced enhancement of influenza immunity is mediated in part by improvements in psychosocial factors in older adults. Brain Behav. Immun..

[13] Woods J.A., Keylock K.T., Lowder T., Vieira V.J., Zelkovich W., Dumich S., Colantuano K., Lyons K., Leifheit K., Cook M. (2009). Cardiovascular exercise training extends influenza vaccine seroprotection in sedentary older adults: The immune function intervention trial. J. Am. Geriatr. Soc..

[14] Ranadive S.M., Cook M., Kappus R.M., Yan H., Lane A.D., Woods J.A., Wilund K.R., Lwamoto G.A., Vanar V., Tandon R. (2014). Effect of acute aerobic exercise on vaccine efficacy in older adults. Med. Sci. Sports Exerc..

[15] Long J.E., Ring C., Drayson M., Bosch J.A., Campbell J., Bhabra J., Browne D., Dawson J., Harding S., Lau J. (2012). Vaccination response following aerobic exercise: Can a brisk walk enhance antibody response to pneumococcal and influenza vaccinations?. Brain Behav. Immun..

[16] Chatterji S., Byles J., Cutler D., Seeman T.E., Verdes E. (2015). Health, functioning, and disability in older adults—Present status and future implications. Lancet.

[17] Suzman R., Beard J.R., Boerma T., Chatterji S. (2015). Health in an ageing world—What do we know?. Lancet.

[18] Senchina D.S., Kohut M.L. (2007). Immunological outcomes of exercise in older adults. Clin. Interv. Aging.

[19] Walford R.L. (1964). The immunologic theory of aging. Gerontologist.

[20] Nichol K.L. (2005). Influenza vaccination in the elderly: Impact on hospitalisation and mortality. Drugs Aging.

[21] Falsey A.R., Walsh E.E. (2005). Respiratory syncytial virus infection in elderly adults. Drugs Aging.

[22] Bender B.S. (2003). Infectious disease risk in the elderly. Immunol. Allergy Clin. N. Am..

[23] High K.P., Bradley S., Loeb M., Palmer R.M., Quagliarello V., Yoshikawa T.T. (2005). A new paradigm for clinical investigation of infectious syndromes in older adults: Assessment of functional status as a risk factor and outcome measure. Clin. Infect. Dis..

[24] Baird R. (2018). Systematic reviews and meta-analytic techniques. Semin. Pediatr. Surg..

[25] Bohn-Goldbaum E., Pascoe A., Singh M.A.F., Singh N.A., Kok J., Dwyer D.E., Mathieson E., Booy R., Edwards K.M. (2020). Acute exercise decreases vaccine reactions following influenza vaccination among older adults. Brain Behav. Immun. Health.

[26] Dangour A.D., Allbala C., Allen E., Grundy E., Walker D., Aedo C., Sanchez H., Fletcher O., Elbourne D., Uauy R. (2011). Effect of a nutrition supplement and physical activity program on pneumonia and walking capacity in chilean older people: A factorial cluster randomized trial. PLoS Med..

[27] Edwards K.M., Pascoe A.R., Fiatarone-Singh M.A., Singh N.A., Kok J., Booy R. (2015). A randomised controlled trial of resistance exercise prior to administration of influenza vaccination in older adults. Brain Behav. Immun..

[28] Hayney M.S., Coe C.L., Muller D., Obasi C.N., Backonja U., Ewers T., Barrett B. (2014). Age and psychological influences on immune responses to trivalent inactivated influenza vaccine in the meditation or exercise for preventing acute respiratory infection (MEPARI) trial. Hum. Vaccines Immunother..

[29] Matsumoto D., Takatori K., Nishida M., Matsushita S. (2015). Effects of home-based exercise with expiratory muscle training on the prevention of falls and aspiration pneumonia in community-dwelling older adults. Physiotherapy.

[30] Takatori K., Matsumoto D., Nishida M., Matsushita S., Noda T., Imamura T. (2016). Benefits of a novel concept of home-based exercise with the aim of preventing aspiration pneumonia and falls in frail older women: A pragmatic controlled trial. BMJ Open Sport Exerc. Med..

[31] Vieira V., Keylock K.T., Lowder T., Zelkovich W., Dumich S., Colantuano K., Potter K., Leifheit K., Mcauley E., Woods J.A. (2008). Effects of exercise training on the immune response to influenza vaccination in older adults: A randomized controlled trial. Brain Behav. Immun..

[32] Yang Y., Verkuilen J., Rosengren K.S., Mariani R.A., Reed M., Grubisich S.A., Woods J.A. (2007). Effects of a Taiji and Qigong intervention on the antibody response to influenza vaccine in older adults. Am. J. Chin. Med..

[33] De Araújo A.L., Silva L.C., Fernandes J.R., De Sousa Toledo Matias M., Boas L.S.V., Machado C.M., Garcezleme L.E., Benard G. (2015). Elderly men with moderate and intense training lifestyle present sustained higher antibody responses to influenza vaccine. Age.

[34] Keylock K.T., Lowder T.W., Leifheit K., Cook M.D., Mariani R.A., Ross K.M., Kim K., Chapmannovakofski K., Mcauley E., Woods J.A. (2007). Higher antibody, but not cell-mediated, responses to vaccination in high physically fit elderly. J. Appl. Physiol..

[35] Kohut M.L., Cooper M.M., Nickolaus M.S., Russell D.R., Cunnick J.E. (2002). Exercise and psychosocial factors modulate immunity to influenza vaccine in elderly individuals. J. Gerontol. Ser. A Biol. Sci. Med. Sci..

[36] Moehling K.K., Nowalk M.P., Lin C.J., Bertolet M., Ross T.M., Carter C.E., Susick M., Saul S., Kaynar A.M., Bromberger J.T. (2018). The effect of frailty on HAI response to influenza vaccine among community-dwelling adults ≥50 years of age. Hum. Vaccines Immunother..

[37] Schuler P.B., Leblanc P.A., Marzilli T.S. (2003). Effect of physical activity on the production of specific antibody in response to the 1998-99 influenza virus vaccine in older adults. J. Sports Med. Phys. Fit..

[38] Wong C.M., Lai H.K., Ou C.Q., Ho S.Y., Chan K.P., Thach T., Yang L., Chau Y.K., Lam T.H., Hedley A.J. (2008). Is exercise protective against influenza-associated mortality?. PLoS ONE.

[39] Yao X., Hamilton R.G., Weng N., Xue Q., Bream J.H., Li H., Tian J., Yeh S., Resnick B., Xu X. (2011). Frailty is associated with impairment of vaccine-induced antibody response and increase in post-vaccination influenza infection in community-dwelling older adults. Vaccine.

[40] Fried L.P., Tangen C.M., Walston J.D., Newman A.B., Hirsch C.H., Gottdiener J., Seeman T.E., Tracy R.P., Kop W.J., Burke B.G. (2001). Frailty in older adults: Evidence for a phenotype. J. Gerontol. Ser. A Biol. Sci. Med. Sci..

[41] Pascoe A.R., Singh M.A.F., Edwards K.M. (2014). The effects of exercise on vaccination responses: A review of chronic and acute exercise interventions in humans. Brain Behav. Immun..

[42] Edwards K.M., Burns V.E., Adkins A.E., Carroll D., Drayson M.T., Ring C. (2008). Meningococcal A vaccination response is enhanced by acute stress in men. Psychosom. Med..

[43] Edwards K.M., Burns V.E., Allen L.M., Mcphee J.S., Bosch J.A., Carroll D., Drayson M.T., Ring C. (2007). Eccentric exercise as an adjuvant to influenza vaccination in humans. Brain Behav. Immun..

[44] Edwards K.M., Burns V.E., Reynolds T., Carroll D., Drayson M.T., Ring C. (2006). Acute stress exposure prior to influenza vaccination enhances antibody response in women. Brain Behav. Immun..

[45] Edwards K.M., Pung M.A., Tomfohr L.M., Ziegler M.G., Campbell J., Drayson M.T., Mills P.J. (2012). Acute exercise enhancement of pneumococcal vaccination response: A randomised controlled trial of weaker and stronger immune response. Vaccine.

[46] Morley J.E., Vellas B., Van Kan G.A., Anker S.D., Bauer J.M., Bernabei R., Cesari M., Chumlea W.C., Doehner W., Evans J. (2013). Frailty consensus: A call to action. J. Am. Med. Dir. Assoc..

[47] Hotfiel T., Carl H.D., Wendler F., Jendrissek A., Heiß R., Swoboda B. (2016). Plantar pressures increase with raising body weight: A standardised approach with paired sample using neutral shoes. J. Back Musculoskelet. Rehabil..

[48] Jian Y., Winter D.A., Ishac M.G., Gilchrist L. (1993). Trajectory of the body COG and COP during initiation and termination of gait. Gait Posture.

[49] O’kane F.W., Mcgibbon C.A., Krebs D.E. (2003). Kinetic analysis of planned gait termination in healthy subjects and patients with balance disorders. Gait Posture.

[50] Perry S.D., Radtke A., Goodwin C.R. (2007). Influence of footwear midsole material hardness on dynamic balance control during unexpected gait termination. Gait Posture.

[51] Keijsers N.L.W., Stolwijk N.M., Pataky T.C. (2010). Linear dependence of peak, mean, and pressure–time integral values in plantar pressure images. Gait Posture.

[52] Kilgore K. (2019). An invitation to live: Insights from an older, long-term practitioner of Tai Chi. Phys. Act. Health.

[53] Ye J., Sun D., Fekete G. (2018). Ba Duan Jin preliminary analysis of the second type of plantar pressure. Phys. Act. Health.

